# Safety of People with Intellectual Disabilities in Hospital. What Can the Hospital Pharmacist Do to Improve Quality of Care?

**DOI:** 10.3390/pharmacy5030044

**Published:** 2017-08-04

**Authors:** Bernadette Flood

**Affiliations:** Daughters of Charity Disability Support Services, D15 DH6F, Dublin, Ireland; bernadette.flood@docservice.ie

**Keywords:** intellectual disabilities, vulnerable, pharmacists, medication, patient safety, hospital, invisibility, patient safety incidents

## Abstract

People with intellectual disabilities are vulnerable in healthcare environments. They experience health and healthcare inequalities, and when admitted to general hospitals are at a greater risk of patient safety incidents. This is well known in specialist services, but less recognized within primary or secondary healthcare. The most significant barriers to safer and better healthcare appear to include ‘invisibility’ of people with intellectual disabilities within health-care systems, widespread lack of staff understanding of intellectual disability, the vulnerabilities of people with intellectual disabilities, and the reasonable adjustment they may need in order to access health-care services. They may be ‘invisible’ to pharmacists in general hospitals. This article aims to raise awareness among those pharmacists and others providing care and support to people with intellectual disabilities in hospital in relation to how pharmacists can contribute to safety. Medication is the main therapeutic intervention in this population. Research is needed to determine the role of pharmacists in improving health outcomes and reducing health inequalities in this vulnerable population group when they are admitted to general hospitals.

## 1. Introduction

People with intellectual disabilities are vulnerable in healthcare environments. They are at particular safety risk in acute hospital settings [1] The care they receive in hospital can be of poor quality. They experience health and healthcare inequities [2] and, on admission to general hospitals, are exposed to more risk of patient safety incidents throughout their care pathway [3]. They have a different health profile from the general population, and their needs are often unrecognised and unmet in general hospitals. This is well known in specialist services but is not so well known within primary or secondary healthcare. There are opportunities for hospital pharmacists to participate in efforts to improve the quality of care provided to people with intellectual disabilities in hospitals.

Professionals and service provider organisations should make changes in their approach or provision of services to ensure that services are accessible to people with disabilities as well as everybody else. Reasonable adjustments to the provision of pharmaceutical care and pharmacy services can mean alterations to buildings by providing wide doors, and accessible information, but may also mean changes to policies, procedures and staff training to ensure that pharmacy services work equally well for people with intellectual disabilities. For example, people with intellectual disabilities may require:

- clear, simple, and possibly repeated explanations of what is happening in hospital, and of treatments and medicines they are prescribed;
- help with participating in meetings with professionals and communicating their thoughts and questions; and
- help with managing issues of consent to medication.

Many hospital staff may be unaware of the needs of people with intellectual disabilities, and this increases their vulnerability [4]. The lack of reasonable adjustments can lead to delays and failures of care, and these can result in harm in the population with intellectual disabilities [5]. Medication use is the main therapeutic intervention in the population with intellectual disabilities. This article aims to remind the hospital pharmacist of the vulnerabilities of people with intellectual disabilities, and to indicate ways in which hospital pharmacists may contribute to efforts to improve the quality of care provided to people with intellectual disabilities while they are in hospital. The author has seventeen years of experience working as a pharmacist in long-term care for people ageing with intellectual disabilities in Ireland, and presented a poster on this topic at the Hospital Pharmacists Association of Ireland Annual Educational Conference in 2014 and the All Ireland Pharmacy Conference in 2015.

## 2. Background

Intellectual disability has its onset before the age of 18 years, and is associated with lifetime limitations in IQ (70 or less) and adaptive functioning. Between 1% to 3% of the world’s population have an intellectual disability [2]. People with intellectual disabilities have a different pattern of illness and mortality from the general population. They have greater healthcare needs and have illnesses that may not be diagnosed or managed correctly. They make significant use of hospital services [6]. Each person with an intellectual disability will have unique and individual needs concerning medication use while in hospital. There is a need to ensure the quality of care at particular points of interaction along the patient journey through a hospitalisation: admission, diagnostic testing, placement on a ward, medication use and discharge. Medication prescribing, dispensing, administration and monitoring are points of interaction with staff of relevance to the person with an intellectual disability, their carer, pharmacists and other clinicians. Disability awareness should be increased among all pharmacists. Improving the awareness of disability on the part of pharmacy staff will help to avoid a situation where health needs are not identified or met, or where ‘diagnostic overshadowing’ occurs and the health problems of a person with an intellectual disability are viewed as part of the person’s condition [7].

The need to provide equal access to healthcare has been highlighted in this vulnerable population [8,9], along with the requirement to raise awareness among healthcare professionals of the health needs of people with disabilities. In particular, pharmacists have a duty of care to the most vulnerable in society who must be treated with dignity by all clinicians and healthcare staff. People with intellectual disabilities need equitable pharmaceutical care and equal outcomes from their interactions with pharmacists in all clinical settings. Coordination and quality are aspects of care that are key components of the provision of accessible services. The reported experiences of people with intellectual disabilities themselves suggest that pharmacists and others

- should never make assumptions about a person’s abilities, preferences, expectations or wishes;
- should ‘just ask them’ how they can help a person with an intellectual disability and about what they prefer and expect; and
- must respect and comply with their expressed preferences.

The United Nations Convention on the Rights of Persons with Disabilities (Article 25) sets out the right of people with disabilities to attain the highest standard of healthcare and nondiscrimination [10]. However, the most significant barriers to safer and better healthcare appear to include ‘invisibility’ of people with intellectual disabilities within health-care systems, widespread lack of staff understanding of intellectual disability, the vulnerabilities of people with intellectual disabilities, and the reasonable adjustment they may need in order to access health-care services.

Research into the safety of patients with intellectual disabilities in NHS hospitals noted delays and omissions in treatment and basic care [11]. People with intellectual disabilities must become visible to hospital pharmacists.

Factors affecting the implementation of strategies to promote a safer environment for patients with intellectual disabilities in hospitals have been identified and are of interest to pharmacists [11]:

- Invisibility of people with intellectual disabilities in hospitals;
- Poor staff understanding of specific vulnerabilities of people with intellectual disabilities;
- Lack of consistent and effective carer involvement; and
- Lack of clear lines of responsibility.

Guidelines on Caring for People with a Learning (intellectual) Disability in General Hospital have been published in Northern Ireland [4], and set out areas for improvement in relation to the person’s journey through a general hospital. A significant number of the concerns identified in the guidelines can be addressed by using better individual care planning, enhanced communication and effective liaison within and between services, and within existing resources. A resource pack, The Pharmaceutical Care of People with Learning Disabilities 2014, has been published by NHS Education for Scotland (Pharmacy). This valuable resource is designed to assist pharmacists and pharmacy technicians, whatever setting they work in, to deliver high-quality pharmaceutical care to people with intellectual/learning disabilities, their relatives and carers. It highlights for pharmacy staff the specific health needs of people with intellectual/learning disabilities and provides additional sources of useful information.

## 3. Support for People with Intellectual Disabilities in Hospital

People with intellectual disabilities make use of hospital services for medical and surgical interventions. Emergency access may also be required, either as a result of a deterioration in a chronic condition such as epilepsy, diabetes, respiratory disease or a gastrointestinal disorder or, as the result of an accident [12].

Family members, carers and support persons play vitally important roles in caring for and supporting people with intellectual disabilities. Their involvement can improve outcomes for the person with an intellectual disability especially if involved in planned admission or discharge from hospital and in helping the person with an intellectual disability to manage a health problem and medication use in the community. Hospital staff do not always understand the essential role of carers and the importance of including carer expertise. There is a risk of compromised patient care when carers of people with intellectual disabilities are not listened to [11].

In primary care, specific areas of support for a person with intellectual disability will include the involvement and value of families, carers and support persons in planning for a person’s accessibility, managing the person’s holistic care, navigating the complex healthcare system, understanding and managing a person’s medication regime, supporting a person with a disability to attend a healthcare appointment, and support with communications. The person with an intellectual disability must be asked for her consent in sharing information or involving her family member, carer or support person.

Pharmacists in all healthcare settings may lack appropriate information about the pharmaceutical needs of people with intellectual disabilities. Maire O’Dwyer and her colleagues undertook a narrative review of the literature to explore what type of pharmaceutical care interventions were being undertaken for people with intellectual disabilities, and how pharmacists contributed to the care of people with intellectual disabilities [13]. The authors concluded that the limited evidence available in the literature suggests that pharmacists can make positive interventions in relation to the quality of the medication use process. Cooperation with carers and patients with intellectual disabilities and other healthcare professionals will be crucial in efforts to improve the quality of the medication use process. There is a recognised need for research to increase the evidence base for the benefits of providing pharmaceutical care to patients with intellectual disabilities and to inform future policy and planning.

## 4. Medication Management

Many of the causes of intellectual disabilities may also lead to physical or mental ill health. This means that people with intellectual disabilities may be more likely to be prescribed multiple medications due to complex and multiple health needs, which, in turn, can sometimes adversely affect health through side effects and drug interactions. Safety incidents can result from delays or omissions of care, including medication prescribing, dispensing and administration in general hospital.

Delays and omissions have been related to failures to implement reasonable adjustments for people with intellectual disabilities, a lack of understanding of ‘consent’, failures to listen to carers, staff perceptions and knowledge of intellectual disabilities, and the characteristics of patients with intellectual disabilities that may make them more vulnerable to safety incidents [11]. Hospital services, particularly accident and emergency departments, are used more frequently by people with intellectual disabilities than by other people. This applies in particular to conditions that are more appropriately treated elsewhere (such as out-patient or primary care clinics), and their stays are long [14,15].

The identification and ‘flagging’ of patients with intellectual disabilities by hospital pharmacies ensures that pharmacists are in a position to implement adequate reasonable adjustments for people with intellectual disabilities who need them. Medication-related safety incidents, in particular, put people with intellectual disabilities at risk of avoidable harm in hospitals. There is a requirement to reconcile all medications when a patient is admitted to and discharged from hospital [16]. This practice is relevant to any patient on medication prior to admission to hospital. It has a greater significance for population groups known for multiple medication use, such as older people, those with intellectual disabilities and those with chronic health conditions. People with intellectual disabilities require an emphasis on this practice at the point of admission to ensure all medications are documented and available at admission and safely administered during their time in hospital [6]. Medication reconciliation would help eliminate safety risks arising from changes in dosages and presentations or delays in medication administration during a period spent in hospital.

Omissions of medication have been identified as a common patient safety issue when people with intellectual disabilities are admitted to hospital. Some examples of situations involving medication management are provided by Tuffrey-Wijne [5] and colleagues:

- Care staff observing the patient’s medication untaken at the bedside. On investigation, the medication was documented as having been administered.
- Nonadministration of medication by hospital staff, not challenged by the person with intellectual disability.
- Persons with intellectual disability are unlikely to challenge an error or medication safety incident thus increasing their vulnerability.
- Medication omissions could be related to patients’ compliance/ non-compliance with treatment.
- Nursing staff working in general hospitals may be unfamiliar with certain medications commonly taken by people with intellectual disabilities, e.g., anti-epileptics and mood stabilisers and lack understanding of the importance of such medications in the person’s care plan.
- Problems have arisen in medication use post discharge from hospital.
- Inadequate transfer of information to carers about medication changes.
- Discharge medication not in an appropriate format for the person.

Recent literature in the area of intellectual disabilities has indicated that there may be no further need for research into the situation of people with intellectual disabilities in hospitals. Iacono and colleagues [6] recognise that addressing medication safety for people with disabilities is problematic, and suggest that efforts to improve medication management should be aligned with existing hospital safety processes. Pharmacists can help reduce health and healthcare inequalities in the population with intellectual disabilities [17]. Medication is the main therapeutic intervention in this population. The complexity of the needs of patients with intellectual disabilities, their multiple medication use and the limited exposure of staff to this group of complex patients may require intellectual disability liaison or ‘specialist’ pharmacists in general hospitals [18].

### Pharmacy

**Figure 1:**
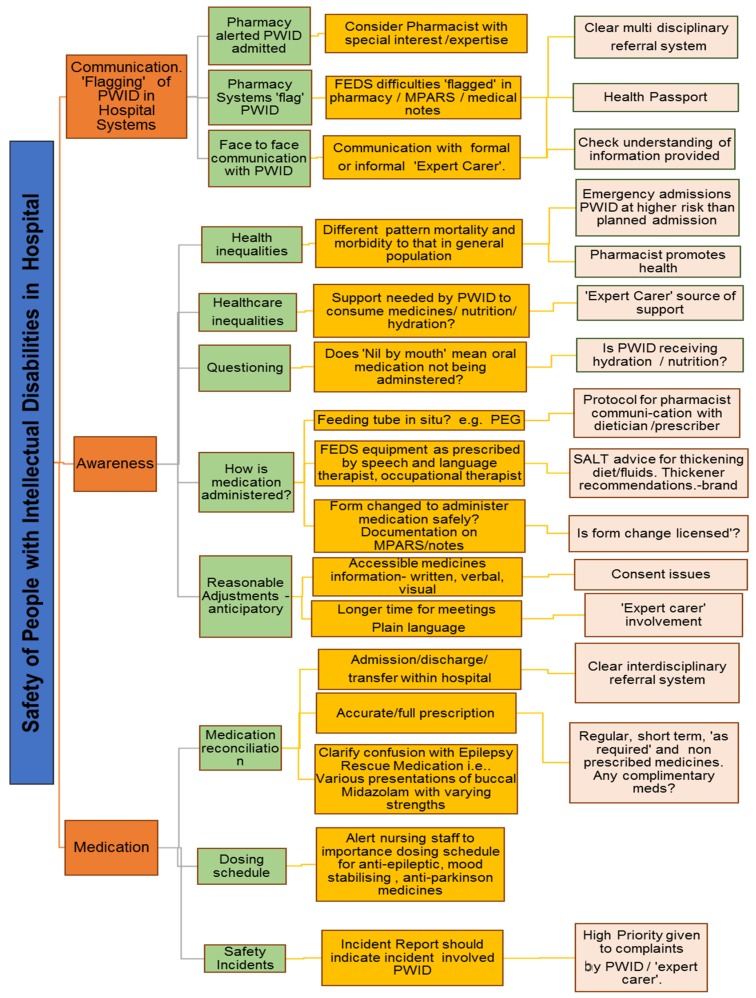
Safety of people with intellectual disabilities in hospital and the pharmacist. PWID = Person with intellectual disability. MPARS = Medication Administration & Recording System. SALT = Speech and Language Therapist. PEG = Percutaneous endoscopic gastrostomy. FEDS = Feeding, eating, drinking or swallowing.

The following chart, safety of people with intellectual disabilities in hospital and the pharmacist Figure 1, is a first attempt to draw the attention of hospital pharmacists to a variety of areas that could be targeted to ensure people with intellectual disabilities receive reasonably adjusted quality care while they are in hospital. Firstly, pharmacists need to know that a patient has an intellectual disability in order to provide quality care. Does the hospital computer system ‘flag’ that the patient has an intellectual disability? Has the pharmacist access to accessible information resources, for example, easy read literature. Does the pharmacist have extra time to communicate with the patient with an intellectual disability and/or their carer? A Mencap report highlighted frequent failings in care such as ignoring vital advice from families and an inability of staff to meet basic care needs [19]. Will the pharmacist accept that the ‘expert’ carer is a valuable resource?

Pharmacy staff need to be aware of both general healthcare and specific medication-related issues. Does the pharmacist know that the prevalence rate of epilepsy amongst people with intellectual disabilities has been reported as at least twenty times higher than for the general population, that uncontrolled epilepsy can have serious negative consequences on both quality of life and mortality, and that the management of epilepsy is particularly important because of the risk of sudden unexpected death in epilepsy (SUDEP)? They need to ask relevant questions. Does ‘Nil by mouth’ mean that oral medications (including, anti-epileptic drugs) are not being administered? Has the patient a clear, up to date prescription and administration protocol for epilepsy rescue medication? What is the patient’s swallowing ability? Has the patient or carer indicated that any form change is needed to administer oral medication? Is any form change safe and licensed? Has the patient had a recent assessment by a qualified speech and language therapist?

Transitions of care are particularly vulnerable for people with intellectual disabilities and people with intellectual disabilities require equitable care that is appropriate for their needs. Does the patient have a ‘Health Passport’ and what is the patient’s preferred method of communication? Can the person consent to treatment?

## 5. Conclusions

Research will be needed to determine the role of pharmacists in improving health outcomes and reducing health inequalities in this vulnerable population group when they are admitted to general hospitals. There is no quick way to improve the quality of care for people with intellectual disabilities while in general hospitals. Innovations in care by many healthcare professions, including pharmacy, will be required. The following areas will require attention:

- Professional pharmacy practice: requires increased awareness of barriers to healthcare, awareness of specific healthcare needs, and addressing of negative and discriminatory attitudes. Opportunities exist for continuing professional development to include the promotion by pharmacists of equal access to health and equitable access to healthcare for people with intellectual disabilities.
- Professional pharmacy education: the vulnerabilities of people with intellectual disabilities should be highlighted during in-service, undergraduate and continuing education programmes. Positive attitudes, communication skills and improving competence can be facilitated by directly involving people with intellectual disabilities in education programmes for pharmacists.
- Policy formulation: pharmacists should facilitate the identification of people with intellectual disabilities in wider healthcare policies and targeted policies. The introduction of a Health Impact Assessment [20], or similar, to assess the impact of a proposed policy on the health of people with intellectual disabilities would be of value.
- Research and data collection: people with intellectual disabilities should be included in public health and pharmacy related surveys, and the role of support workers/families in promoting access to healthcare and quality medication use should be valued and understood.

This article is an attempt to raise awareness of the patient with an intellectual disability among hospital pharmacists. Hospital pharmacy management will need to target resources and improve the awareness of all pharmacy staff to address accessibility and the poorer experiences of people with intellectual disabilities in general hospitals. Individual hospital pharmacists will need to take responsibility for vulnerable patients in their care. Ensuring quality care is provided by pharmacy departments and individual pharmacists to the most vulnerable patients facilitates the provision of quality care for all.
